# Synthesis of Optically Active Poly(diphenylacetylene)s Using Polymer Reactions and an Evaluation of Their Chiral Recognition Abilities as Chiral Stationary Phases for HPLC

**DOI:** 10.3390/molecules21111487

**Published:** 2016-11-07

**Authors:** Katsuhiro Maeda, Miyuki Maruta, Yuki Sakai, Tomoyuki Ikai, Shigeyoshi Kanoh

**Affiliations:** Graduate School of Natural Science and Technology, Kanazawa University, Kakuma-machi, Kanazawa 920-1192, Japan; amyrtt@gmail.com (M.M.); y.sakai.kanazawa.u@gmail.com (Y.S.); ikai@se.kanazawa-u.ac.jp (T.I.); kanoh@se.kanazawa-u.ac.jp (S.K.)

**Keywords:** high-performance liquid chromatography (HPLC), chiral stationary phase, enantioseparation, poly(diphenylacetylene), helix, helix inversion

## Abstract

A series of optically active poly(diphenylacetylene) derivatives bearing a chiral substituent (poly-**2***S*) or chiral and achiral substituents (poly-(**2***S*_x_-*co*-**3**_1−x_)) on all of their pendant phenyl rings were synthesized by the reaction of poly(bis(4-carboxyphenyl)acetylene) with (*S*)-1-phenylethylamine ((*S*)-**2**) or benzylamine (**3**) in the presence of a condensing reagent. Their chiroptical properties and chiral recognition abilities as chiral stationary phases (CSPs) for high-performance liquid chromatography (HPLC) were investigated. Poly-**2***S* and poly-(**2***S*_x_-*co*-**3**_1−x_) (0.06 < x < 0.71) formed a preferred-handed helical conformation with opposite helical senses after thermal annealing despite possessing the same chiral pendant (*h*-poly-**2***S* and *h*-poly-(**2***S*_x_-*co*-**3**_1−x_)). Furthermore, *h*-poly-**2***S* and *h*-poly-(**2***S*_0.36_-*co*-**3**_0.64_) emitted circularly polarized luminescence with opposite signs. *h*-Poly-**2***S* showed higher chiral recognition abilities toward a larger number of racemates than poly-**2***S* without a preferred-handed helicity and the previously reported preferred-handed poly(diphenylacetylene) derivative bearing the same chiral substituent on half of its pendant phenyl rings. *h*-Poly-(**2***S*_0.36_-*co*-**3**_0.64_) also exhibited good chiral recognition abilities toward several racemates, though the elution order of some enantiomers was reversed compared with *h*-poly-**2***S*.

## 1. Introduction

It is well known that a pair of enantiomers often show significant differences in their physiological activities. The development of efficient techniques for the separation of enantiomers is therefore important in several fields, especially in the pharmaceutical industry. The direct separation of enantiomers by high-performance liquid chromatography (HPLC) using a chiral stationary phase (CSP) is currently one of the most popular and effective methods for both analyzing the composition of enantiomeric mixtures and purifying such mixtures to give pure enantiomers [1,2,3,4,5,6]. However, the success of this method is entirely dependent on the development of effective CSPs with excellent resolving abilities. A large number of optically active small molecules [7,8,9,10,11] and polymers [1,2,3,12,13,14,15] have been evaluated to date as CSPs for HPLC. However, the development of novel CSPs with excellent resolving abilities is still highly desired because there are still several chiral compounds that cannot be resolved using existing commercially available CSPs.

**Figure 1:**
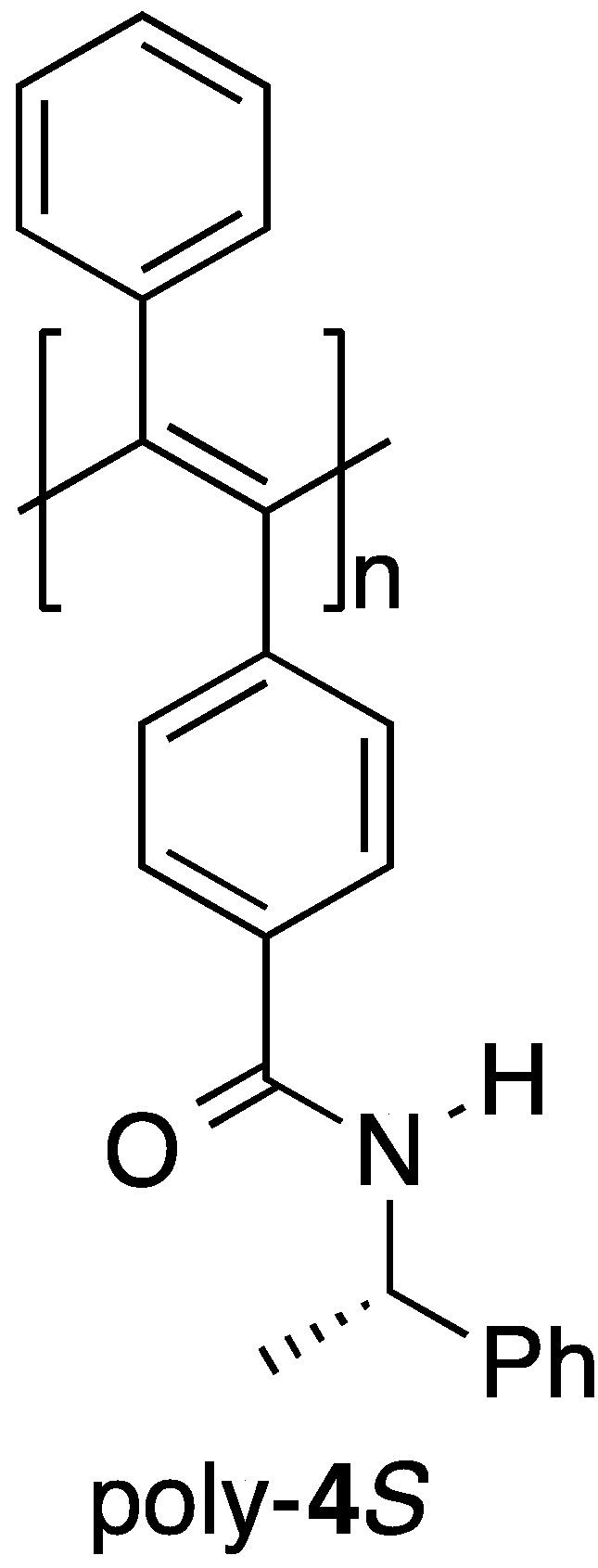
The structure of poly-**4***S*.

Optically active poly(phenylacetylene)s with a preferred-handed helicity have been prepared [16] and some of these systems have been reported to exhibit good chiral recognition abilities as CSPs for HPLC because of their preferred-handed helical conformation [17,18,19,20,21,22,23,24,25,26]. However, very few optically active poly(diphenylacetylene)s have been prepared to date, which has limited research towards evaluating the scope and efficiency of the chiral recognition abilities of these materials [27,28,29,30,31,32,33,34,35]. We recently reported the first example of an optically active poly(diphenylacetylene)-based CSP for HPLC [36]. In this particular case, we synthesized an optically active poly(diphenylacetylene) bearing a chiral pendant amide moiety (poly-**4***S*) (Figure 1) by the reaction of an optically inactive precursor polymer bearing a pendant carboxyl group with the optically active amine (*S*)-1-phenylethylamine (*S*)-**2**. The results revealed that this polymer showed good chiral recognition ability towards various racemates when it was used as a CSP for HPLC and that its chiral recognition ability was significantly dependent on its preferred-handed helical conformation. Notably, the preferred-handed helical conformation of the polymer was induced by the thermal annealing process (*h*-poly-**4***S*), which was applied after the introduction of the optically active pendants via a polymer reaction. The chiral recognition abilities of *h*-poly-**4***S* with a preferred-handed helicity were greater than those of poly-**4***S* without a preferred-handed helicity. This result indicated that the macromolecular helicity induced in the polymer backbone by thermal annealing as a consequence of the effect of the chiral pendant groups was playing an important role in the high chiral recognition ability of this material. This finding therefore inspired us to synthesize several other optically active poly(diphenylacetylene)s with the aim of developing more practically useful poly(diphenylacetylene)-based CSPs for HPLC. Although poly(diphenylacetylene)s have pendant phenyl rings on all of the carbons in their main chain, only half of the pendent phenyl rings in poly-**4***S* feature an amide functional group, which could act as effective chiral recognition sites. In this study, we designed and synthesized several novel optically active poly(diphenylacetylene)s bearing chiral or achiral substituents, which were connected to the pendant phenyl rings of the polymer through an amide bond (poly-**2***S* and poly-(**2***S*_x_-*co*-**3**_1–x_)). We also investigated the chiroptical properties and chiral recognition abilities of these materials as CSPs for HPLC.

## 2. Results and Discussion

**Scheme 1:**
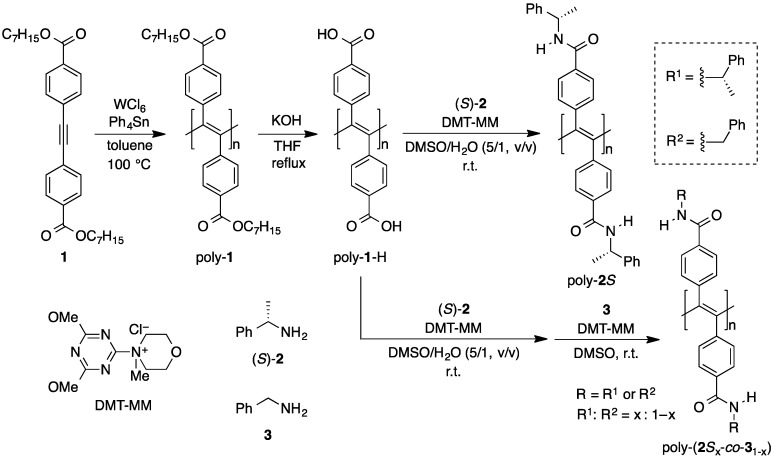
Synthesis of poly-**2***S* and poly-(**2***S*_x_-*co*-**3**_1−x_).

The synthetic route used to prepare poly-**2***S* and poly-(**2***S*_x_-*co*-**3**_1–x_) is shown in Scheme 1. The direct polymerization of diphenylacetylene monomers bearing polar functional groups, such as carboxyl and amide groups, hardly proceeds because the group 5 transition metals used for polymerization of disubstituted acetylenes are intolerant to such polar functional groups [37,38]. With this in mind, the carboxyl groups of the diphenylacetylene monomer (**1**) were protected as the corresponding *n*-heptyl esters prior to being polymerized in toluene with WCl_6_–Ph_4_Sn at 100 °C. Notably, these conditions have been reported to be effective for the polymerization of diphenylacetylenes bearing ester groups [32,39]. It was also envisaged that the long alkyl (*n*-heptyl) chains of the ester groups would enhance the solubility of the resulting polymer in toluene. The polymerization reaction of **1** proceeded smoothly to give poly-**1** with a moderate molecular weight (*M*_n_ = 1.3 × 10^4^, *M*_w_/*M*_n_ = 1.6) in high yield (77%). The ester groups in poly-**1** were then hydrolyzed under alkaline conditions to give poly-**1**-H. The complete removal of the *n*-heptyl esters was confirmed by ^1^H-NMR and IR spectroscopy (Figures S1 and S2). Poly-**2***S* was prepared in a similar manner to that previously reported for poly-**4***S* [36], by the polymer reaction of poly-**1**-H with (*S*)-**2** using 4-(4,6-dimethoxy-1,3,5-triazin-2-yl)-4-methylmorpholinium chloride (DMT-MM) as a condensing reagent. The near complete introduction of the (*S*)-**2** residue was confirmed by ^1^H NMR, IR and elemental analyses (Figures S1 and S3). Poly-(**2***S*_x_-*co*-**3**_1–x_)s were synthesized by the two-step polymer reaction of poly-**1**-H with (*S*)-**2**, followed by benzylamine (**3**) using DMT-MM as a condensing reagent. Poly-**1**-H was initially reacted with (*S*)-**2** in the presence of a controlled amount of DMT-MM to allow for the introduction of a small number of (*S*)-**2** units as the pendant groups. The resulting material was then reacted with an excess of **3** in the presence of an excess of DMT-MM. The (*S*)-**2** unit contents (x) of the resulting poly-(**2***S*_x_-*co*-**3**_1–x_)s were determined by ^1^H-NMR analysis (see Figure S3). Poly-(**2***S*_x_-*co*-**3**_1–x_)s containing different amounts of the (*S*)-**2** units were obtained by changing the feed ratio of DMT-MM relative to poly-**1**-H whilst maintaining that of (*S*)-**2** relative to DMT-MM ([(*S*)-**2**]/[DMT-MM] = 2) (Table S1). The (*S*)-**2** unit contents of the resulting polymers were found to correspond to approximately one-third of an equivalent of the DMT-MM feed in the first step.

**Figure 2:**
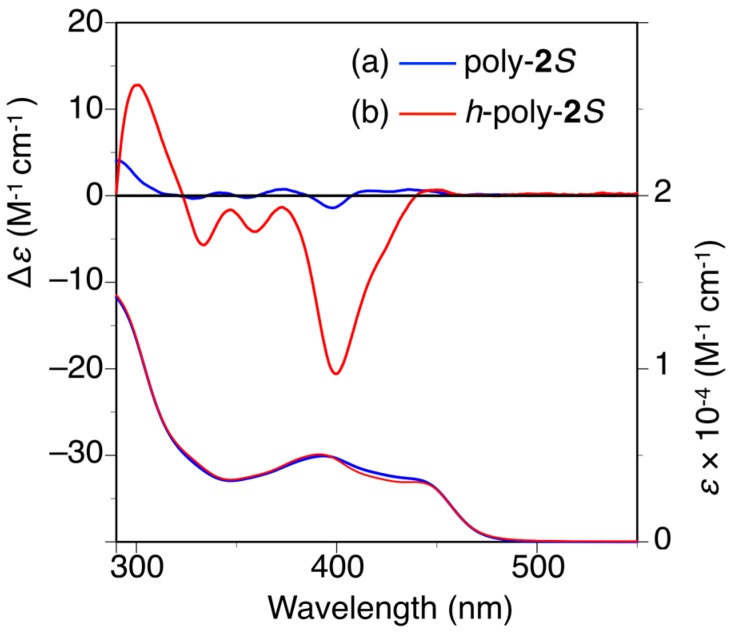
CD and absorption spectra of poly-**2***S* (**a**) and *h*-poly-**2***S* (**b**) in *N*,*N*-dimethylformamide (DMF) at 25 °C. *h*-Poly-**2***S* was obtained by annealing poly-**2***S* in DMF at 120 °C for 2 h.

The circular dichroism (CD) spectrum of poly-**2***S* showed almost no circular dichroism (CD) in the wavelength region longer than 300 nm ascribed to the absorption of the polymer backbone in *N*,*N*-dimethylformamide (DMF) immediately after the preparation of the sample (Figure 2a) [27,32,33,34,35]. However, an apparent Cotton effect was observed around 400 nm after one day. Furthermore, the CD intensity very slowly increased with time in DMF at 25 °C, but it did not reach a constant value even after 8 days (Figure S4a). When the solution was annealed at higher temperatures, the CD intensity rapidly increased to reach a plateau (Δ*ε*_400_ = ca. −21) after 5 h at 90 °C or 2 h at 120 °C, which was accompanied by a negligible change in the absorption spectra (Figure 2b and Figures S4b and S5). These results indicated that a predominantly one-handed helical conformation was induced in the polymer backbone by thermal annealing owing to the effect of the optically active pendants (*h*-poly-**2***S*). A similar thermal annealing was necessary for poly-**4***S* to form the preferred-handed helical conformation (*h*-poly-**4***S*) [27,36]. This result was attributed to steric hindrance between the neighboring pendant phenyl rings, which was large enough to prevent poly-**2***S* and poly-**4***S* from being transformed into their preferred-handed helical conformations after the introduction of the optically active pendants.

**Figure 3:**
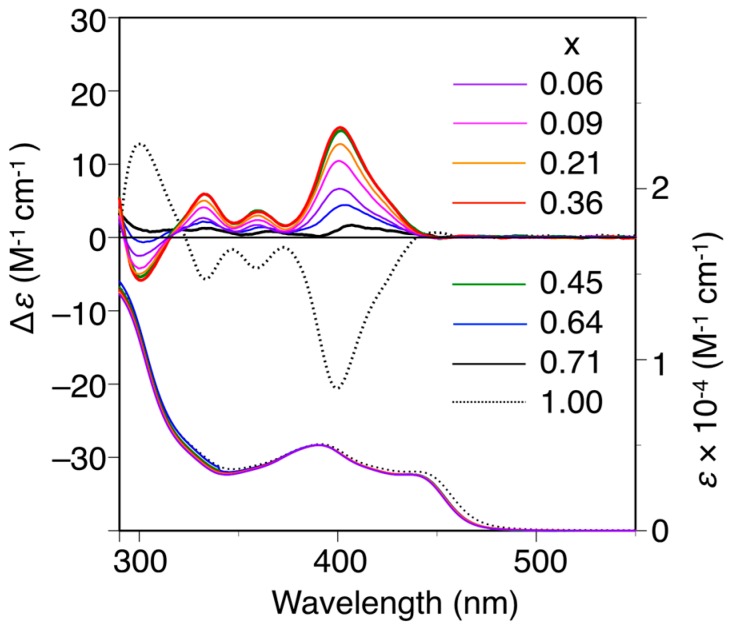
CD and absorption spectra of poly-(**2***S*_x_-*co*-**3**_1–x_) and *h*-poly-**2***S* in DMF at 25 °C. *h*-poly-(**2***S*_x_-*co*-**3**_1–x_) and *h*-poly-**2** were obtained by annealing poly-(**2***S*_x_-*co*-**3**_1–x_) and poly-**2***S*, respectively, in DMF at 120 °C for 2 h.

**Figure 4:**
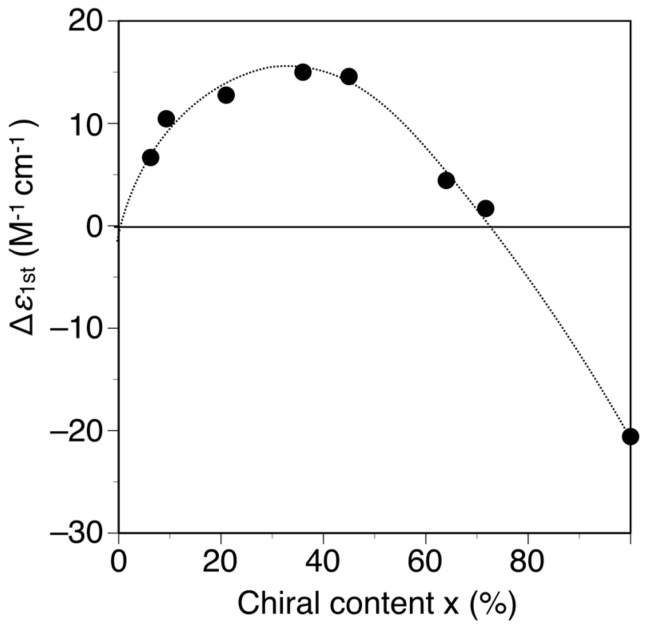
Plots of the Δ*ε*_1st_ values of *h*-poly-(**2***S*_x_-*co*-**3**_1–x_) and *h*-poly-**2***S* in DMF at 25 °C vs. their chiral (*S*)-**2** unit contents (x).

The poly-(**2***S*_x_-*co*-**3**_1−x_)s were converted to the corresponding *h*-poly-(**2***S*_x_-*co*-**3**_1−x_)s with predominantly one-handed helical conformations in the same way by annealing the corresponding DMF solutions at 120 °C for 2 h. Figure 3 shows the CD and absorption spectra of the *h*-poly-(**2***S*_x_-*co*-**3**_1–x_)s in DMF at 25 °C together with those of *h*-poly-**2***S*. The CD intensities of these polymers (Δ*ε* of the first Cotton effect) were plotted against their chiral (*S*)-**2** unit contents, as shown in Figure 4. Interestingly, the Cotton effects of the *h*-poly-(**2***S*_x_-*co*-**3**_1–x_)s gave the opposite sign to that of *h*-poly-**2***S* when the (*S*)-**2** content was below ca. 71 mol %. Furthermore, the CD spectrum of *h*-poly-(**2***S*_0.36_-*co*-**3**_0.64_) was almost a mirror image of that of *h*-poly-**2***S* accompanied with a negligible change in the absorption. These results indicated that the poly-(**2***S*_x_-*co*-**3**_1–x_)s (x < 0.71) existed in a predominantly one-handed helical conformation with an opposite helical-sense to that of *h*-poly-**2***S*. This system therefore allowed for the selective formation of right- and left-handed helical structures using a single enantiomer of **2** as the chiral unit, with the actual outcome being dependent on the mole fraction of **2**. Similar helical sense inversion behaviors in the copolymers consisting of chiral and achiral units depending on the contents of the chiral units have been also reported in polyisocyanates [40], polysilanes [41], polyacetylenes [42,43,44], polyisocyanides [45] and poly(quinoxaline-2,3-diyl)s [46]. It has also been reported that these phenomena can be explained using a modified version of the Ising model proposed by Sato et al., where the helix-sense is determined by the interactions between the neighboring pendant groups, chiral–chiral units and chiral–achiral units, which can stabilize the opposite helix-sense to each other [47].

**Figure 5:**
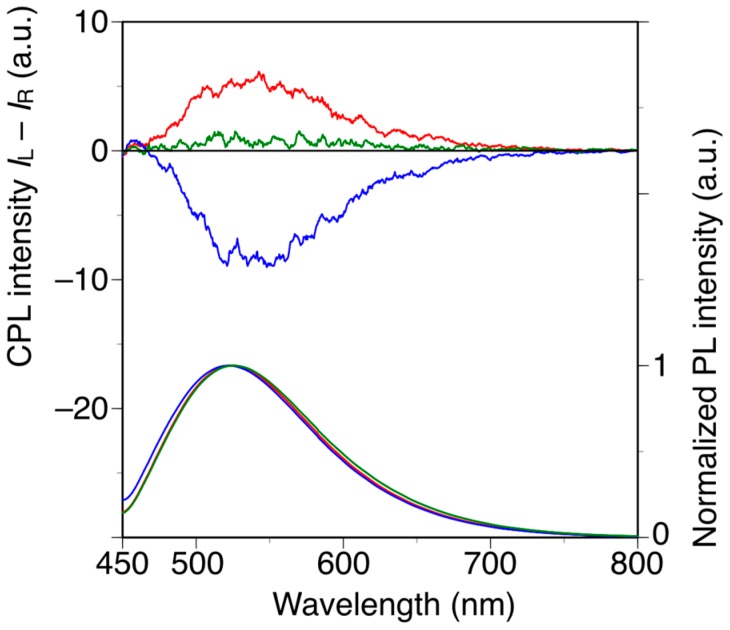
CPL and PL spectra of *h*-poly-**2** (blue line), *h*-poly-(**2***S*_0.36_-*co*-**3**_0.64_) (red line) and poly-(**2***S*_0.36_-*co*-**3**_0.64_) (green line) in DMF at 25 °C with excitation at 388 nm. [polymer] = 1.0 × 10^−5^ M.

Poly(diphenylacetylene)s are highly emissive without the introduction of any other fluorescent substituents, whereas poly(phenylacetylene)s are generally non-emissive materials [38,48]. With this in mind, we investigated the relationship between the main-chain conformation and the circularly polarized luminescence (CPL) properties of *h*-poly-**2***S* and *h*-poly-(**2***S*_0.36_-*co*-**3**_0.64_). The CPL and photoluminescence (PL) spectra of *h*-poly-**2***S*, *h*-poly-(**2***S*_0.36_-*co*-**3**_0.64_) and poly-(**2***S*_0.36_-*co*-**3**_0.64_) in DMF are shown in Figure 5. Photographic images of these solutions under irradiation at 365 nm are shown in Figure S6. *h*-Poly-**2***S* and *h*-poly-(**2***S*_0.36_-*co*-**3**_0.64_) showed equal and opposite CPL bands around 520 nm, which were consistent with the corresponding PL bands. In contrast, no apparent CPL signal was observed for poly-(**2***S*_0.36_-*co*-**3**_0.64_). The signs of the CPL signals at 520 nm were identical to those of the CD band around 400 nm and the values of the emission dissymmetry factor (*g*_lum_), which is defined as *g*_lum_ = 2(*I*_L_ − *I*_R_)/(*I*_L_ + *I*_R_), where *I*_L_ and *I*_R_ are the intensities of the left- and right-handed circularly polarized light, respectively, were estimated to be 1.2 × 10^−3^ for *h*-poly-**2***S* and 0.8 × 10^−3^ for *h*-poly-(**2***S*_0.36_-*co*-**3**_0.64_). These results indicated that *h*-poly-**2***S* and *h*-poly-(**2***S*_0.36_-*co*-**3**_0.64_) can emit opposite CPL originating from the preferred-handed helical conformation with opposite helical sense to each other despite having the same central chirality in their pendants.

**Figure 6:**
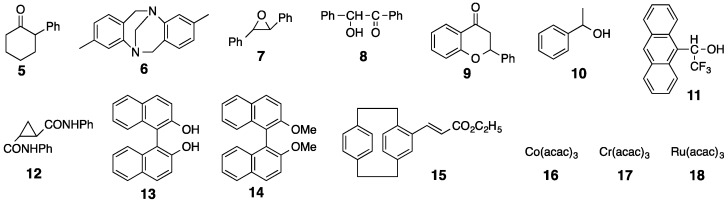
Structures of racemates **5**–**18**.

**Figure 7:**
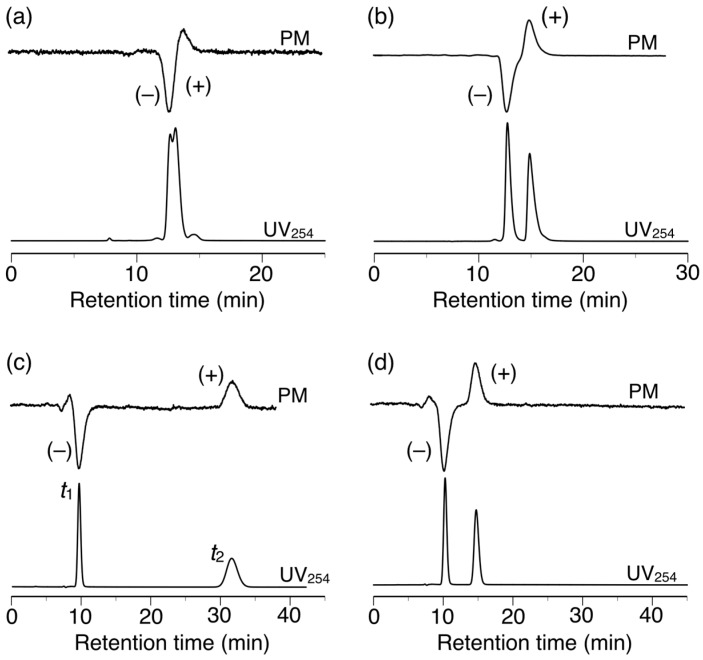
HPLC chromatograms for the resolution of **15** (**a**,**b**) and **16** (**c**,**d**) on poly-**2***S* (**a**,**c**) and *h*-poly-**2***S* (**b**,**d**). Eluent: hexane–2-propanol (99:1, *v*/*v*) (**a**,**b**), MeOH–H_2_O (90:10, *v*/*v*).

The chiral recognition abilities of poly-**2***S*, *h*-poly-**2***S* and *h*-poly-(**2***S*_0.36_-*co*-**3**_0.64_) as CSPs for HPLC were evaluated using the various racemic compounds with different functional groups (**5**–**12**) including axial (**13**, **14**) or planar (**15**) chiral compounds as well as chiral metal complexes (**16**–**18**) (Figure 6). The packing materials were prepared by coating macroporous silica gel (particle size 7 μm, pore size 100 nm) with DMF solutions of the corresponding polymers [49]. The obtained packing materials were subsequently packed into a stainless steel column (25 × 0.20 cm (i.d.)) using a conventional slurry method [50]. The chromatographic resolution results are summarized in Table 1 and the chromatograms obtained for the resolutions of racemic **15** and **16** on poly-**2***S*- and *h*-poly-**2***S*-based CSPs are shown in Figure 7. Ultraviolet (UV) and polarimetric (PM) detectors were used for the detection and identification of the peaks, respectively. As shown in Figure 7c, the different enantiomers of **16** were eluted with retention times of *t*_1_ ((–)-enantiomer) and *t*_2_ ((+)-enantiomer), which showed a complete baseline separation on the poly-**2***S*-based CSP. The retention factor *k*_1_ (*k*_1_ = (*t*_1_ − *t*_0_)/*t*_0_) for the first eluted enantiomer, the separation factor *α* (*α* = (*t*_2_ − *t*_0_)/(*t*_1_ − *t*_0_)) and the resolution factor *R*_S_ (*R*_S_ = 2(*t*_2_ − *t*_1_)/(*w*_1_ + *w*_2_)), where *t*_0_ is the hold-up time and *w*_1_ and *w*_2_ are the band widths of the first- and second-eluted enantiomers on the base line, were determined to be 0.14, 19.5 and 10.2, respectively.

Poly-**2***S* partially or completely resolved nine of the 14 racemates tested in the current study. Notably, poly-**2***S* exhibited excellent chiral recognition abilities toward chiral metal acetylacetonate complexes **16**–**18**, showing complete baseline separations in all three cases, together with very high *α* values (*α* > 15) (Figure 7c). Methanol/H_2_O (90:10, *v*/*v*) was used as the eluent because the enantiomers of **16**–**18** were not eluted with hexane-2-propanol.

*h*-Poly-**2***S* showed higher chiral recognition ability than poly-**2***S* toward seven racemates. For example, *h*-poly-**2***S* resolved **15** with complete baseline separation (Figure 7b), whereas poly-**2***S* only achieved a partial separation of the same compound (Figure 7a). Moreover, *h*-poly-**2***S* resolved racemates **8** and **9**, which were barely separated on poly-**2***S*. These results therefore indicated that the preferred-handed helical conformation induced in *h*-poly-**2***S* was playing an important role in its excellent chiral recognition ability, which was consistent with the results of our previous report for *h*-poly-**4***S* [36]. As expected, poly-**2***S* and *h*-poly-**2***S* both exhibited higher enantioselectivities toward nine of the racemates than our previously reported poly-**4***S* and *h*-poly-**4***S* systems, respectively, bearing the same (*S*)-**2** unit on half of their phenyl pendants [36]. This result could be attributed to an increase in the number of the amide pendants acting as effective chiral recognition sites compared with the previous systems. In our previous study, we also observed an interesting inversion in the order of the enantioseparation of the chiral metal complexes **16**–**18** on the poly-**4***S*- and *h*-poly-**4***S*-based CSPs, despite the fact that they had the same optically active groups of their pendant moieties [36]. This phenomenon can be explained by the opposite enantioselectivity between the central chirality in the optically active pendants and the induced macromolecular helicity of the polymer backbone toward these racemates, which would represent a negative synergetic effect. In contrast, *h*-poly-**2***S* and poly-**2***S* completely resolved racemates **16**–**18** with good enantioselectivities (*α* = 2.9–3.6), but the elution order was the same on both CSPs (Figure 7c,d). The effect of the induced macromolecular helicity of *h*-poly-**2***S* on the separation of these racemates therefore appeared to have been cancelled out by the central chirality of the pendants, because *h*-poly-**2***S* had twice as many chiral pendants as *h*-poly-**4***S*.

**Figure 8:**
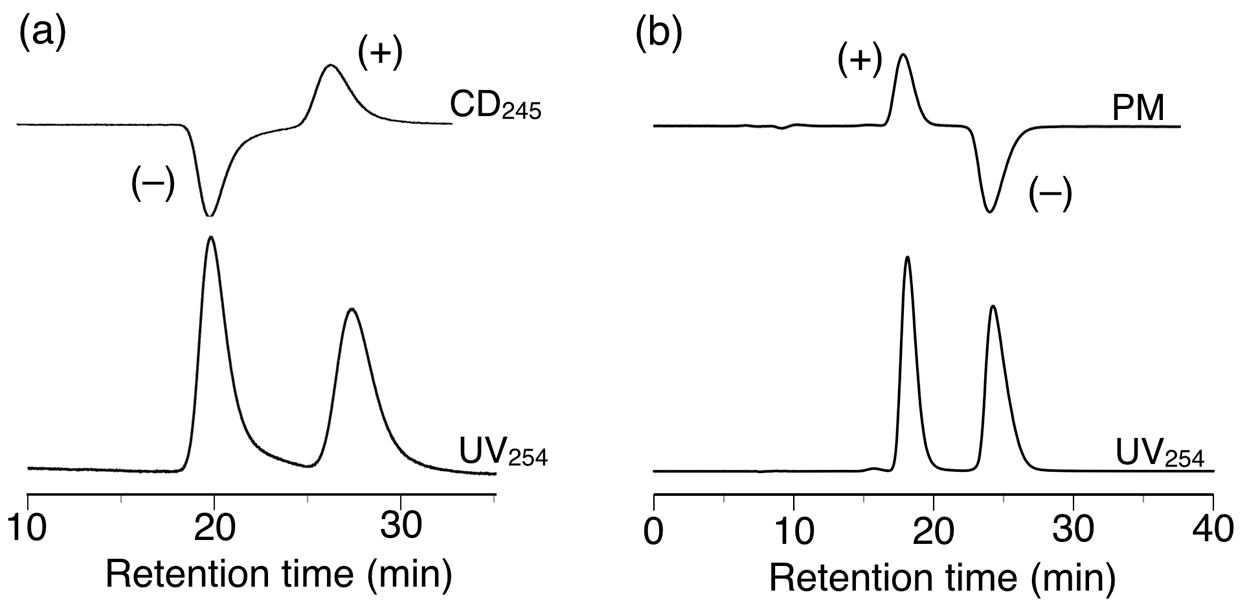
HPLC chromatograms for the resolution of **14** (**a**) and **15** (**b**) on *h*-poly-(**2***S*_0.36_-*co*-**3**_0.64_). Eluent: hexane–2-propanol (99:1, *v*/*v*).

*h*-Poly-(**2***S*_0.36_-*co*-**3**_0.64_) exhibited different chiral recognition properties to *h*-poly-**2***S* and successfully resolved 11 of the 14 racemates evaluated in the current study. It is noteworthy that *h*-poly-(**2***S*_0.36_-*co*-**3**_0.64_) resolved racemates **6** and **14**, which were not resolved by poly-**2***S* or *h*-poly-**2***S* (Table 1 and Figure 8a). Among the enantiomers separated using *h*-poly-(**2***S*_0.36_-*co*-**3**_0.64_), the elution orders of six of the racemates (**5**–**7**, **9**, **13** and **15**) were the opposite of those achieved on *h*-poly-**2***S*. In particular, the complete baseline separation of the racemic cyclophane derivative **15** with a planar chirality was achieved on *h*-poly-**2***S* and *h*-poly-(**2***S*_0.36_-*co*-**3**_0.64_) with a completely reversed elution order; with the (–)-enantiomer eluting prior to the (+)-enantiomer on *h*-poly-**2***S* (*α* = 1.38) (Figure 7b) and vice versa on *h*-poly-(**2***S*_0.36_-*co*-**3**_0.64_) (*α* = 1.56) (Figure 8b). These results clearly suggested that the macromolecular helicity induced in these polymers was making a major contribution to the resolution of these racemates.

## 3. Materials and Methods

### 3.1. Reagents and Materials

Copper(I) iodide (CuI) and tungsten(VI) chloride (WCl_6_) were purchased from Kanto Kagaku (Tokyo, Japan) and Aldrich (Milwaukee, WI, USA), respectively. Bis(triphenylphosphine)palladium(II) dichloride (Pd(PPh_3_)_2_Cl_2_) and tetraphenyltin (Ph_4_Sn) were purchased from Tokyo Kasei (TCI, Tokyo, Japan). Triphenylphosphine (PPh_3_) and **3** were purchased from Wako Chemicals (Osaka, Japan). Porous spherical silica gel with a mean particle size of 7 μm and a mean pore diameter of 100 nm (Daiso gel SP-1000-7), trimethylsilylacetylene and (*S*)-**2** were kindly provided by Daiso Chemical (Osaka, Japan), Shin-Etsu Chemical (Tokyo, Japan) and Yamakawa Chemical (Tokyo, Japan), respectively. Anhydrous tetrahydrofuran (THF), toluene, dichloromethane, *N*,*N*-dimethylformamide (DMF) and pyridine were obtained from Kanto Kagaku. Anhydrous dimethyl sulfoxide (DMSO) was purchased from Aldrich. Triethylamine (TEA) was dried over KOH pellets and distilled onto KOH under nitrogen. These solvents were subsequently stored under nitrogen before being used. Heptyl 4-iodobenzoate [36] and 4-(4,6-dimethoxy-1,3,5-triazin-2-yl)-4-methylmorpholinium chloride (DMT-MM) [51] were prepared according to the previously reported methods.

### 3.2. Instruments

Melting points were measured on a Yanako melting point apparatus (Yanako, Kyoto, Japan) and reported as the uncorrected values. NMR spectra were recorded on a JNM-ECA 500 (JEOL, Tokyo, Japan) (500 MHz for ^1^H, 125 MHz for ^13^C) spectrometer using CDCl_3_, DMSO-*d*_6_ or DMSO-*d*_6_-D_2_O as the solvent. TMS was used as an internal reference for the samples run in CDCl_3_ (^1^H and ^13^C), whereas the residual solvent peaks were used as internal reference peaks for the samples run in DMSO-*d*_6_ and DMSO-*d*_6_-D_2_O (^1^H and ^13^C). IR spectra were recorded with a JASCO Fourier Transform IR-460 spectrophotometer (JASCO, Hachioji, Japan). Absorption and CD spectra were measured in a 1.0 mm quartz cell on a JASCO V-650 spectrophotometer and a JASCO J-725 spectropolarimeter, respectively. The temperature was controlled with a JASCO PTC-348WI apparatus. The polymer concentration was calculated based on the monomer units. The CPL and PL spectra were recorded on a JASCO CPL-300 spectrometer and a JASCO FP-6300 spectrofluorometer, respectively, at room temperature. Size exclusion chromatography (SEC) measurements were performed on a JASCO PU-2080 liquid chromatograph equipped with a UV-vis (JASCO UV-970) detector at 40 °C using a Shodex (Tokyo, Japan) KF-805F SEC column. The temperature was controlled with a JASCO CO-1560 column oven. THF was used as the eluent at a flow rate of 1.0 mL/min. Molecular weight calibration curves were generated using polystyrene standards (Tosoh, Tokyo, Japan). The enantiomers were separated by chromatography using a JASCO PU-2080 liquid chromatograph equipped with a multiwavelength detector (JASCO MD-2018) and a polarimetric detector (JASCO OR-990, Hg without filter) or a CD detector (JASCO CD-2095) at ambient temperature. Elemental analyses were performed at the Research Institute for Instrumental Analysis of Advanced Science Research Center, Kanazawa University, Kanazawa, Japan.

### 3.3. Synthesis of Monomer

Bis(4-heptyloxycarbonylphenyl)acetylene (**1**) was prepared according to the route shown in Scheme S1.

*Synthesis of heptyl 4-((trimethylsilyl)ethynyl)benzoate*: To a solution of heptyl 4-iodobenzoate (10.1 g, 29.3 mmol) in anhydrous TEA (50 mL) was added trimethylsilylacetylene (4.30 mL, 31.7 mmol), Ph_3_P (126 mg, 0.480 mmol), CuI (135 mg, 0.192 mmol) and Pd(PPh_3_)_2_Cl_2_ (81.8 mg, 0.117 mmol), and the resulting solution was stirred at room temperature for 20 h. The reaction mixture was filtered through Celite, and the filtrate was evaporated to dryness to give a residue, which was purified by column chromatography over silica gel eluting with a 1:30 (*v*/*v*) mixture of ethyl acetate and hexane to give the desired product as a yellow oil (9.23 g, 98% yield). ^1^H-NMR (500 MHz, CDCl_3_, r.t.): δ 7.96 (d, *J* = 8.0 Hz, 2H, Ar–H), 7.51 (d, *J* = 8.0 Hz, 2H, Ar–H), 4.30 (t, *J* = 6.6 Hz, 2H, OC*H*_2_CH_2_), 1.76 (quint, *J* = 6.6 Hz, 2H, OCH_2_C*H*_2_), 1.25–1.46 (m, 8H, 4CH_2_), 0.89 (t, *J* = 6.6 Hz, 3H, CH_3_), 0.26 (s, 9H, TMS).

*Synthesis of heptyl 4-ethynylbenzoate*: To a solution of heptyl 4-((trimethylsilyl)ethynyl)benzoate (5.96 g, 18.8 mmol) in a 3:1 (*v*/*v*) mixture of THF and methanol (360 mL) was added K_2_CO_3_ (12.9 g, 93.6 mmol), and the resulting mixture was stirred at room temperature for 1 h. The suspension was then filtered and the filtrate was concentrated under reduced pressure. The concentrated solution was diluted with ethyl acetate and washed sequentially with aqueous 1 N HCl and saturated NaHCO_3_ solution, before being dried over Na_2_SO_4_. The solvent was removed by evaporation to give the crude product as a residue, which was purified by column chromatography over silica gel eluting with a 1:30 (*v*/*v*) mixture of ethyl acetate and hexane to give the desired product as a colorless oil (3.56 g, 77% yield). ^1^H-NMR (500 MHz, CDCl_3_, r.t.): δ 7.99 (d, *J* = 8.6 Hz, 2H, Ar–H), 7.55 (d, *J* = 8.0 Hz, 2H, Ar–H), 4.31 (t, *J* = 6.6 Hz, 2H, OC*H*_2_CH_2_), 3.22 (s, 1H, CH), 1.76 (quint, *J* = 6.6 Hz, 2H, OCH_2_C*H*_2_), 1.26–1.46 (m, 8H, 4CH_2_), 0.89 (t, *J* = 6.9 Hz, 3H, CH_3_).

*Synthesis of bis(4-heptyloxycarbonylphenyl)acetylene* (**1**): To a solution of heptyl 4-iodobenzoate (5.03 g, 14.5 mmol) in anhydrous TEA (25 mL) was added Ph_3_P (57.1 mg, 0.218 mmol), CuI (63.6 mg, 0.334 mmol), Pd(PPh_3_)_2_Cl_2_ (40.8 mg, 0.0581 mmol) and heptyl 4-ethynylbenzoate (3.55 g, 14.5 mmol), and the resulting mixture was stirred at room temperature for 20 h. The reaction mixture was then filtered through Celite and the filtrate was concentrated under reduced pressure to give a residue, which was diluted with ethyl acetate and washed sequentially with water and brine, before being dried over Na_2_SO_4_. The solvent was removed under reduced pressure to give a residue, which was purified by column chromatography over silica gel eluting with a 1:25 (*v*/*v*) mixture of ethyl acetate and hexane to give the crude product. The crude product was subsequently purified by recrystallization from a mixture of toluene and methanol to give the desired product as a white solid (5.11 g, 77% yield). M.p.: 61.3–62.4 °C. IR (KBr, cm^−1^): 1943 (*ν*_C≡C_), 1707 (*ν*_C=O_). ^1^H-NMR (500 MHz, CDCl_3_, r.t.): δ 8.04 (d, *J* = 8.6 Hz, 4H, Ar–H), 7.60 (d, *J* = 8.0 Hz, 4H, Ar–H), 4.33 (t, *J* = 6.6 Hz, 4H, 2OC*H*_2_CH_2_), 1.76 (quint, *J* = 6.6 Hz, 4H, 2OCH_2_C*H*_2_), 1.25–1.47 (m, 16H, 8CH_2_), 0.90 (t, *J* = 6.9 Hz, 6H, 2CH_3_). ^13^C-NMR (125 MHz, CDCl_3_, r.t.): δ 166.19, 131.76, 130.46, 129.68, 127.38, 91.49, 65.56, 31.88, 29.11, 28.85, 26.15, 22.75, 14.23. Elemental analysis: Calcd. for C_30_H_38_O_4_: C, 77.89; H, 8.28. Found: C, 77.60; H, 8.37.

### 3.4. Polymerization

The polymerization reaction was carried out in a Schlenk flask under argon using a WCl_6_-Ph_4_Sn catalyst system according to the literature method [32,36]. Monomer **1** (1.05 g, 2.27 mmol), WCl_6_ (90 mg, 0.23 mmol) and Ph_4_Sn (98 mg, 0.23 mmol) were placed in a Schlenk flask under argon, followed by anhydrous toluene (4.5 mL), which was added using a syringe. The resulting mixture was heated at 100 °C under stirring for 24 h. The reaction mixture was then cooled to room temperature and treated with a large amount of methanol to precipitate the polymer, which was collected by centrifugation and then dried in vacuo at room temperature overnight. The dried polymer was purified by reprecipitation from toluene using a 3:1 (*v*/*v*) mixture of methanol and THF as an antisolvent system, and the resulting solid was dried in vacuo at room temperature overnight to give poly-**1**-Hep (0.81 g, 77% yield). The molecular weight (*M*_n_) of poly-**1**-Hep was estimated to be 1.3 × 10^4^ (*M*_w_/*M*_n_ = 1.6) by SEC using polystyrene standards with THF as the eluent. IR (KBr, cm^−1^): 1721 (*ν*_C=O_ of ester). ^1^H-NMR (500 MHz, CDCl_3_, 25 °C): δ 7.16–7.28 (br, 4H, Ar–H), 6.41–6.71 (br, 2H, Ar–H), 5.92–6.15 (br, 2H, Ar–H), 4.03–4.48 (br, 4H, 2OC*H*_2_CH_2_), 1.60–1.93 (br, 4H, 2OCH_2_C*H*_2_), 1.25–1.47 (br, 16H, 8CH_2_), 0.79–1.04 (br, 6H, 2CH_3_). ^13^C-NMR (125 MHz, CDCl_3_, 25 °C): δ 165.60, 146.48, 146.33, 130.63, 128.88, 128.34, 126.99, 65.44, 31.90, 29.17, 28.67, 25.98, 22.74, 14.18. Elemental analysis: Calcd. for C_30_H_38_O_4_: C, 77.89; H, 8.28. Found: C, 77.40; H, 8.42.

Poly-**1**-Hep was converted to poly-**1**-H by the hydrolysis of its ester groups as follows. A solution of poly-**1**-Hep (0.71 g) in THF (30 mL) was treated with a 4 N solution of aqueous KOH (25 mL), and the resulting mixture was stirred at 80 °C for 3 h. The mixture was cooled to ambient temperature and the THF was removed under reduced pressure to give a concentrated mixture, which was treated with a 4 N aqueous solution of KOH (25 mL). The resulting mixture was then stirred at 80 °C for 72 h to complete the hydrolysis. The solution was subsequently cooled to ambient temperature and acidified with a 6 N aqueous HCl solution, resulting in the precipitation of poly-**1**-H, which was collected by centrifugation, washed with water and dried in vacuo at room temperature overnight to give the desired product (243 mg, 60% yield). IR (KBr, cm^−1^): 1700 (*ν*_C=O_ of carboxylic acid). ^1^H-NMR (500 MHz, D_2_O/DMSO-*d*_6_ (1/1, *v*/*v*), 25 °C): δ 5.30–8.00 (br, 8H, Ar–H). ^13^C-NMR (125 MHz, D_2_O/DMSO-*d*_6_ (1/2, *v*/*v*), 25 °C): δ 126.99, 128.06, 129.61, 131.18, 147.10, 167.65. Elemental analysis: Calcd. for C_16_H_10_O_4_: C, 72.18; H, 3.79. Found: C, 72.36; H, 3.98.

### 3.5. Synthesis of Poly-***2***S by Using a Polymer Reaction

A solution of poly-**1**-H (150 mg, 0.56 mmol) and (*S*)-**2** (278 μL, 2.2 mmol) in a 5:1 (*v*/*v*) mixture of DMSO and H_2_O (26 mL) was treated with DMT-MM (620 mg, 2.2 mmol), and the resulting mixture was stirred at room temperature for 24 h. The mixture was then added to a large volume of methanol, resulting in the precipitation of the poly-**2***S* product, which was collected by centrifugation and then dried in vacuo at room temperature. The resulting poly-**2***S* product was found to contain approximately 100 mol % of (*S*)-**2** as the pendant group to each monomer unit, as determined by ^1^H-NMR and elemental analyses (Figure S3a).

Spectroscopic data for poly-**2***S*: IR (KBr, cm^−1^): 1635 (*ν*_C=O_ of amide), 1533 (*ν*_N__–H_ of amide). ^1^H-NMR (500 MHz, CDCl_3_, 25 °C): δ 8.40–7.60 (br, 2H, NH), 7.60–4.70 (br, 20H, aromatic, C*H*CH_3_), 2.00–1.10 (br, 6H, CHC*H*_3_). Elemental analysis: Calcd. for C_32_H_28_N_2_O_2_·0.85H_2_O: C, 78.78; H, 6.14; N, 5.74. Found: C, 78.85; H, 5.88; N, 5.84.

### 3.6. Synthesis of Poly-(***2***S_x_-co-***3***_1−x_) by Using Polymer Reactions

Typical procedure for synthesis of poly-(**2***S*_x_-*co*-**3**_1−x_) by polymer reaction of poly-**1**-H with (*S*)-**2** followed by **3** using DMT-MM as a condensing reagent is described below. To a solution of poly-**1**-H (100 mg, 0.37 mmol) and (*S*)-**2** (67 μL, 0.54 mmol) in DMSO (6 mL) was added DMT-MM (75 mg, 0.27 mmol), and the resulting mixture was stirred at room temperature for 12 h. The reaction mixture was then poured into a 5:1 (*v*/*v*) mixture of hexane and ethanol, and the resulting precipitated polymer was collected by centrifugation, washed sequentially with a 1 N aqueous solution of HCl, water and methanol, and then dried under vacuum at room temperature overnight. The dried polymer was dissolved in DMSO (6 mL) and treated with an excess of **3** (200 μL, 1.87 mmol) in the presence of DMT-MM (258 mg, 0.93 mmol) for 12 h at room temperature. The reaction mixture was then poured into a large volume of methanol, and the resulting precipitated polymer collected by centrifugation and dried under vacuum at room temperature overnight to yield poly-(**2***S*_0.36_-*co*-**3**_0.64_) (125 mg, 74%). The (*S*)-**2** content of the polymer was determined to be 36 mol % by ^1^H-NMR analysis (Figure S3b).

Spectroscopic data for poly-(**2***S*_0.36_-*co*-**3**_0.64_): IR (KBr, cm^−1^): 1638 (*ν*_C=O_ of amide), 1523 (*ν*_N__–H_ of amide). ^1^H-NMR (500 MHz, CDCl_3_, 25 °C): δ 8.75–8.02 (br, 2H, NH), 7.66–5.75 (br, 18H, aromatic), 5.27–4.80 (br, C*H*CH_3_), 4.58–3.96 (br, CH_2_), 1.57–0.85 (br, CHC*H*_3_). Calcd. for 0.36C_32_H_28_N_2_O_2_·0.64C_30_H_24_N_2_O_2_·0.6H_2_O: C, 79.27; H, 5.77; N, 6.02. Elemental analysis: Found: C, 79.13; H, 5.53; N, 5.98.

The other poly-(**2***S*_x_-*co*-**3**_1−x_)s were synthesized in the same way by varying the feed ratio of DMT-MM relative to poly-**1**-H whilst maintaining that of (*S*)-**2** against DMT-MM ([(*S*)-**2**]/[DMT-MM] = 2) (see Table S1). The (*S*)-**2** unit contents (x) of the poly-(**2***S*_x_-*co*-**3**_1−x_)s were determined by ^1^H-NMR analysis.

### 3.7. Preparation of High-Performance Liquid Chromatography (HPLC) Columns

A solution of poly-**2**, *h*-poly-**2***S* or *h*-poly-(**2***S*_0.36_-*co*-**3**_0.64_) (100 mg) in DMF (1 mL) was used to coat some macroporous silica gel (400 mg) according to a previously reported method [49], and the solvent was then evaporated under reduced pressure. The poly-**2**, *h*-poly-**2***S* and *h*-poly-(**2***S*_0.36_-*co*-**3**_0.64_) contents on the silica gel were estimated to be 18.3, 17.6 and 19.9 wt %, respectively, by thermogravimetric analysis. After fractionating the packing materials with sieves, the resulting materials were packed into a stainless steel tube (25 × 0.20 cm (i.d.)) using a conventional high-pressure slurry packing technique with an ECONO-PACKER MODEL CPP-085 (Chemco, Osaka, Japan) [50]. The plate numbers for the columns were 3700 (poly-**2***S*), 3500 (*h*-poly-**2***S*) and 3300 (*h*-poly-(**2***S*_0.36_-*co*-**3**_0.64_)), respectively, for the elution of benzene with hexane/2-propanol (90:10, *v*/*v*) at a flow rate of 0.1 mL/min. The hold-up times (*t*_0_) were estimated using 1,3,5-tri-*tert*-butylbenzene and acetone as the non-retained compounds for the normal and reverse phases, respectively [52].

## 4. Conclusions

In conclusion, we have synthesized a series of optically active poly(diphenylacetylene)s bearing amide functional groups on all of their pendant phenyl rings. These systems consist of a chiral unit (poly-**2***S*) or both chiral and achiral units (poly-(**2***S*_x_-*co*-**3**_1–x_)) and were prepared by the reaction of an optically inactive poly(diphenylacetylene) bearing carboxyl groups with chiral or achiral amines. The resulting polymers were evaluated in terms of their chiroptical properties and chiral recognition abilities toward various racemates such as chiral stationary phases (CSPs) for high-performance liquid chromatography (HPLC). We found that poly-**2***S* and poly-(**2***S*_x_-*co*-**3**_1–x_) folded into a preferred-handed helical conformation (*h*-poly-**2***S* and *h*-poly-(**2***S*_x_-*co*-**3**_1–x_)) by thermal annealing. Notably, the helical senses of these materials varied considerably depending on their chiral unit contents. Furthermore, the circular dichroism (CD) and circularly polarized luminescence (CPL) spectra of *h*-poly-**2***S* and *h*-poly-(**2***S*_0.36_-*co*-**3**_0.64_) were almost mirror images of each other. We have demonstrated that these polymers can efficiently resolve various racemates when used as CSPs for HPLC and that the enantioselectivities and elution orders of the enantiomers were significantly influenced by the helical structures induced in these polymers. The elution orders of some enantiomers were reversed on the *h*-poly-**2***S*- and *h*-poly-(**2***S*_0.36_-*co*-**3**_0.64_)-based CSPs because of the opposite helicities induced in these polymers. Moreover, poly-**2***S* and *h*-poly-**2***S* showed higher chiral recognition abilities toward many racemates than the previously reported poly(diphenylacetylene) system bearing the same chiral substituent on only half of its pendant phenyl rings. This result was attributed to an increase in the number of the amide pendants acting as effective chiral recognition sites [36]. Poly(diphenylacetylene)s are thermally and chemically more stable than poly(phenylacetylene)s and are highly emissive without introducing any other fluorescent substituents. We therefore believe that these findings will not only allow for the design of useful poly(diphenylacetylene)-based CSPs for HPLC with much higher chiral recognition abilities toward diverse racemates, but that they will also assist in the development of new circularly polarized luminescence materials.

## Figures and Tables

**Table 1:** Resolution results for racemates **5**–**18** on poly-**2***S*, *h*-poly-**2***S*, *h*-poly-(**2***S*_0.36_-*co*-**3**_0.64_) and *h*-poly-**4***S* ^a^.

Racemates	Poly-2*S*			*h*-poly-2*S*			*h*-Poly-(2*S*_0.36_-*co*-3_0.64_)			*h*-Poly-4*S* ^e^		
	*k*_1_	*α*	*R*_s_	*k*_1_	*α*	*R*_s_	*k*_1_	*α*	*R*_s_	*k*_1_	*α*	*R*_s_
**5**	0.90 (−)	1.17	1.27	1.39 (−)	1.25	1.96	2.81 (+)	1.07	0.90	1.75 (−)	1.06	0.86
**6**	0.47 (−)	ca. 1	—	0.60 (−)	ca. 1	—	1.49 (+)	1.09	0.83	1.16 (−)	1.14	1.06
**7**	0.14 (+)	ca. 1	—	0.12 (+)	ca. 1	—	0.25 (−)	ca. 1	—	0.41 (−)	ca. 1	—
**8**	0.90 (+)	ca. 1	—	0.74 (+)	1.14	1.15	7.19 (+)	1.07	0.95	5.71 (+)	1.13	1.63
**9**	0.90 (+) ^d^	ca. 1	—	0.93 (+) ^d^	1.14	0.92	1.90 (−) ^d^	1.07	0.65	2.14	1.0	—
**10**	3.62 (+)	1.05	0.86	3.24 (+)	ca. 1	—	0.61 (+)	ca. 1	—	0.46 (+)	ca. 1	—
**11**	8.94 (+)	1.13	1.72	12.4 (+)	1.20	2.49	17.6 (+)	1.04	0.50	22.7 (+)	1.16	1.43
**12** ^b^	0.47 (+)	1.44	1.18	0.41 (+)	1.48	1.07	3.84 (+)	ca. 1	—	1.10 (+)	1.51	1.08
**13** ^b^	1.57 (−)	1.30	1.83	1.91 (−)	1.38	1.74	3.56 (+)	1.16	1.13	3.91	1.0	—
**14**	1.06	1.0	—	0.94	1.0	—	4.36 (−)	1.50	3.16	4.02 (−) ^d^	1.98	3.09
**15**	0.74 (−)	1.08	0.78	0.80 (−)	1.38	2.03	1.52 (+)	1.56	2.43	1.45 (−)	1.16	0.92
**16** ^c^	0.14 (−)	19.5	10.2	0.29 (−)	2.94	3.99	7.21 (−)	2.53	6.01	0.18 (+)	2.59	2.09
**17** ^c^	0.39 (+)	15.3	13.5	0.61 (+)	3.00	6.31	9.92 (+)	2.84	6.69	0.28 (−)	2.60	2.88
**18** ^c^	0.64 (−) ^d^	17.5	15.7	0.97 (−) ^d^	3.64	8.88	17.3 (−) ^d^	2.92	7.09	0.45 (+) ^d^	2.38	3.33

^a^ Column: 25 × 0.20 cm (i.d.); Eluent: *n*-hexane/2-propanol (99:1, *v*/*v*); Flow rate: 0.1 mL/min. The signs in parentheses represent the optical rotations of the first-eluted enantiomers; ^b^ Eluent: *n*-hexane/2-propanol (90:10, *v*/*v*); ^c^ Eluent: methanol/H_2_O (90:10, *v*/*v*); ^d^ The signs in parentheses represent the signs of Cotton effects at 254 nm of the first-eluted enantiomers; ^e^ Cited from Reference [36].

## Supplementary Materials

Supplementary materials can be accessed at: http://www.mdpi.com/1420-3049/21/11/1487/s1.
