# Supplementary material for: Synthesis of Optically Active Poly(diphenylacetylene)s Using Polymer Reactions and an Evaluation of Their Chiral Recognition Abilities as Chiral Stationary Phases for HPLC
Source: Molecules. 2016 Nov 7;21(11):1487. doi: 10.3390/molecules21111487 (PMC6273228; doi:10.3390/molecules21111487)
Supplement: Supplementary file 1 [file molecules-21-01487-s001.pdf]

Supplementary Materials: Synthesis of Optically Active Poly(diphenylacetylene)s Using Polymer Reactions and an Evaluation of Their Chiral Recognition Abilities as Chiral Stationary Phases for HPLC

Katsuhiro Maeda, Miyuki Maruta, Yuki Sakai, Tomoyuki Ikai and Shigeyoshi Kanoh

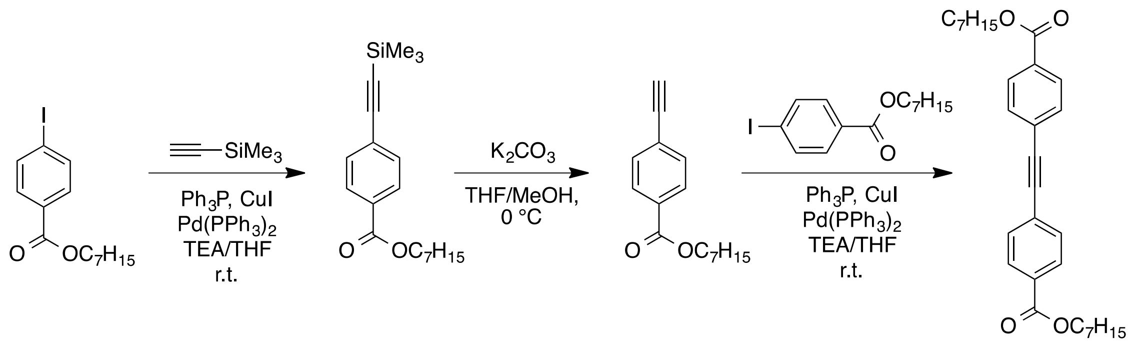

1

Scheme S1. Synthesis of **1**.

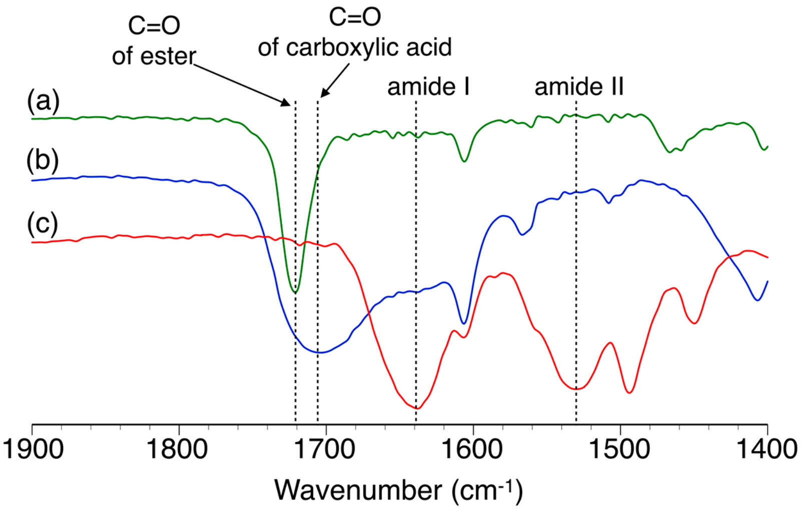

Figure S1. Infrared (IR) spectra of poly-**1** (**a**); poly-**1**-H (**b**) and poly-**2***S* (**c**) as KBr pellets.

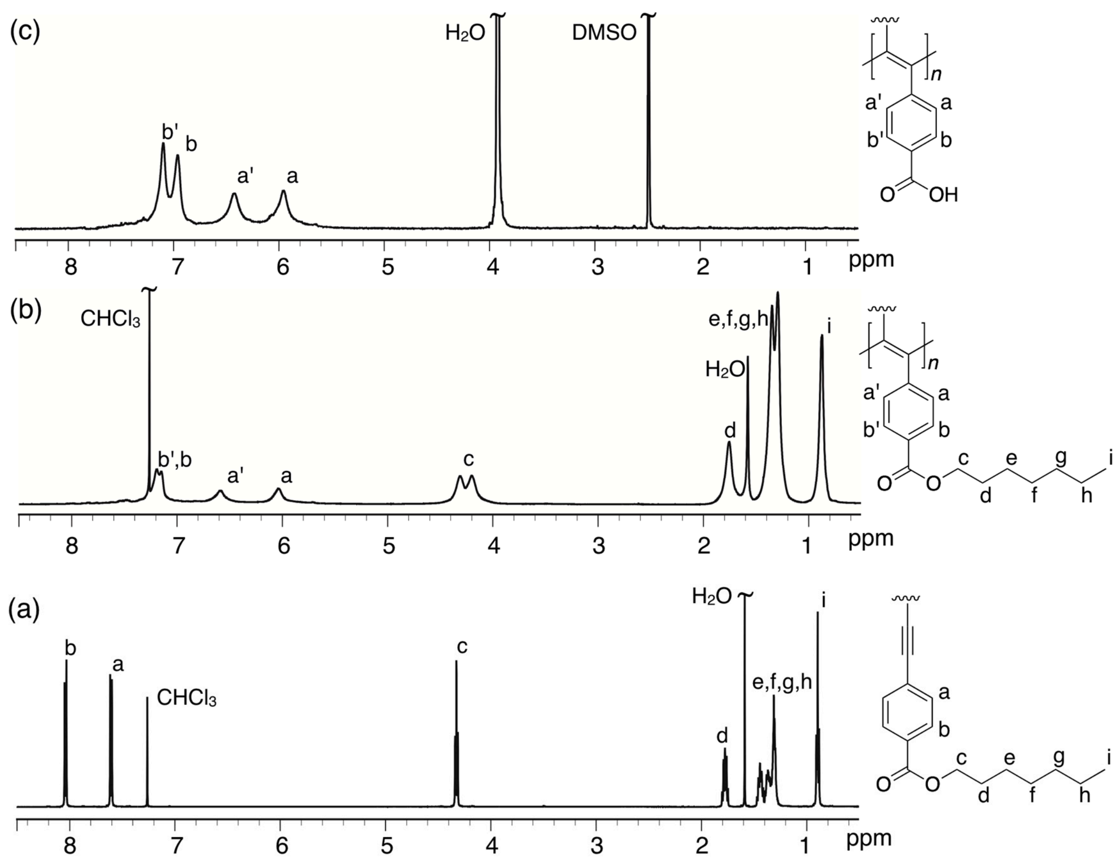

Figure S2. ^{1}H-NMR (500 MHz) spectra of **1** (**a**); poly-**1** (**b**) and poly-**1**-H (**c**) at r.t. These spectra were measured in $CDCl_3$ (**a**,**b**) and DMSO-d_6/D_2O (9/1, *v*/*v*) (**c**).

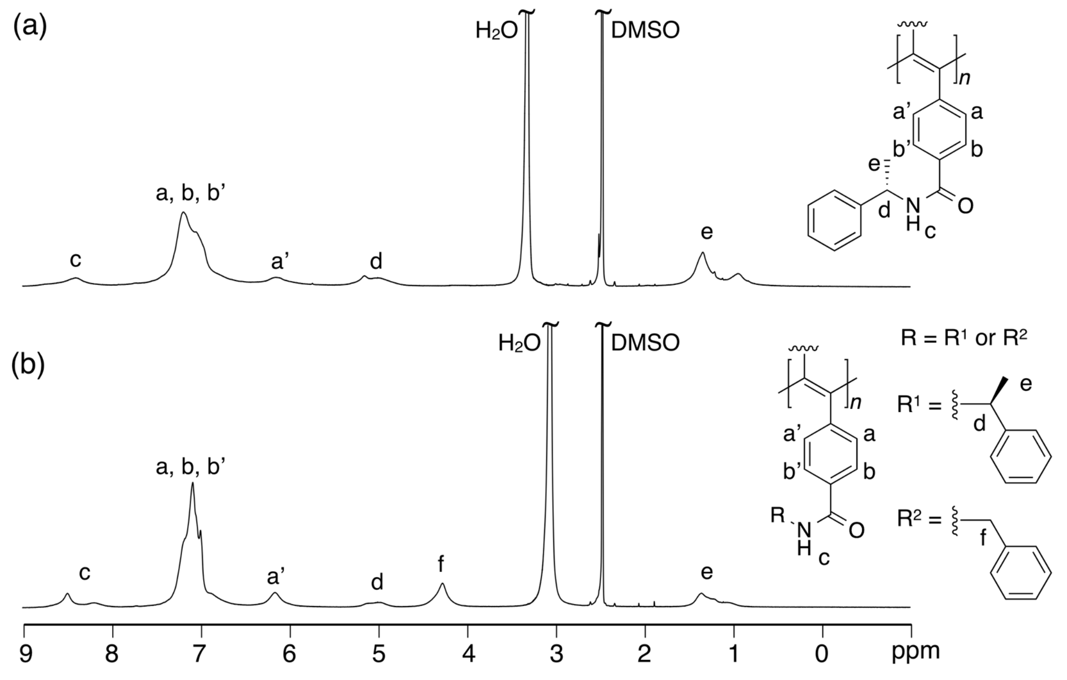

Figure S3. ^{1}H-NMR (500 MHz) spectra of poly-**2***S* (**a**) and poly-(**2***S*$_{0.36}$-*co*-**3**$_{0.64}$) (**b**) in DMSO-d_6 at 25 °C.

Table S1. Synthesis of poly-(**2***S*$_x$-*co*-**3**$_{1-x}$)s by the polymer reaction of poly-**1**-H with (*S*)-**2** followed by the reaction of the product with **3** using DMT-MM as a condensing reagent [a].

Run	[DMT-MM]/[poly-1-H]	[(*S*)-2]/([(*S*)-2] + [3]) in Copolymer (x) [b]	Copolymer [c]
1	2.25	0.71	poly-(**2***S*$_{0.71}$-*co*-**3**$_{0.29}$)
2	1.80	0.64	poly-(**2***S*$_{0.64}$-*co*-**3**$_{0.36}$)
3	1.05	0.45	poly-(**2***S*$_{0.45}$-*co*-**3**$_{0.55}$)
4	0.72	0.36	poly-(**2***S*$_{0.36}$-*co*-**3**$_{0.64}$)
5	0.60	0.21	poly-(**2***S*$_{0.21}$-*co*-**3**$_{0.79}$)
6	0.30	0.09	poly-(**2***S*$_{0.09}$-*co*-**3**$_{0.91}$)
7	0.15	0.06	poly-(**2***S*$_{0.06}$-*co*-**3**$_{0.94}$)

[a] The reactions were carried out in DMSO at room temperature for 12 h. [(*S*)-**2**]/[DMT-MM] = 2, [poly-**1**-H] = 0.05 M (runs 1–3, 5–7) and 0.06 M (run 4). After the reaction with (*S*)-**2**, the resulting polymers were reacted with an excess of **3** in the presence of DMT-MM ([**3**]/[DMT-MM]/[poly-**1**-H] = 4/2.5/1) in DMSO at room temperature for 12 h; [b] Estimated by ^{1}H-NMR; [c] The subscript numbers represent the molar ratios of the corresponding units in the copolymers.

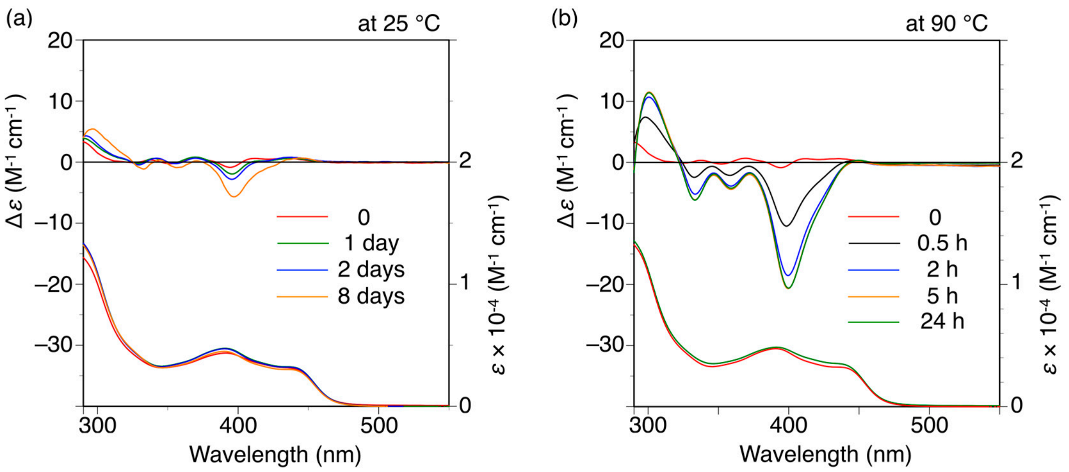

Figure S4. Time-dependent changes in the CD spectra of poly-**2***S* at 25 °C (**a**) and 90 °C (**b**) in DMF.

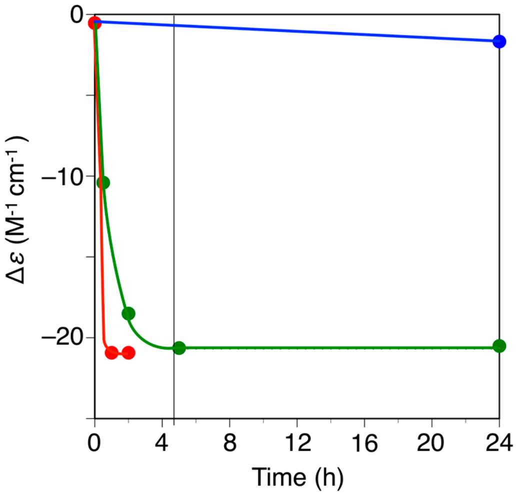

Figure S5. Plots of the CD intensity ($\Delta\varepsilon_{1st}$) of poly-**2***S* at 120 °C (red), 90 °C (green) and 25 °C (blue) in DMF with time.

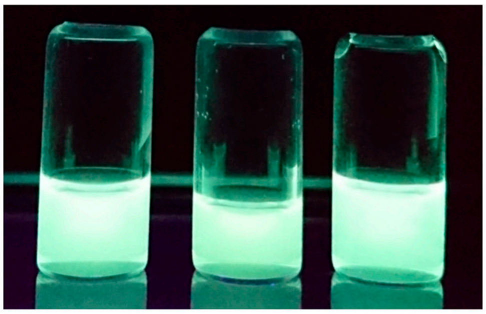

(a) (b) (c)

Figure S6. Photographs of *h*-poly-**2***S* (**a**); *h*-poly-(**2***S*$_{0.36}$-*co*-**3**$_{0.64}$) (**b**) and poly-(**2***S*$_{0.36}$-*co*-**3**$_{0.64}$) (**c**) in DMF at 25 °C under irradiation of 365 nm UV light.
